# A Case of Ruptured Tubal Pregnancy
1Read at the March Meeting of the Bristol Medico-Chirurgical Society.


**Published:** 1897-06

**Authors:** W. H. C. Newnham

**Affiliations:** Physician-Accoucheur, Bristol General Hospital


### A CASE OF RUPTURED TUBAL PREGNANCY.[1]

BY

W. H. C. Newnham, M.A., M.B. Cantab., M.R.C.S. Eng.,

*Physician-Accoucheur, Bristol General Hospital.*

---

For centuries tubal pregnancy was considered one of Nature's rarest freaks, and practitioners of the old school say now, "How is it that we never met with such a case?" I am constantly assailed with some such remark as this, as though it were really possible to make a mistake in the diagnosis. Since March, 1883, when Lawson Tait performed his first successful operation on a case of ruptured tubal pregnancy, examples of this condition have been noticed so frequently that the literature of reported cases is most voluminous. We now know that it is a comparatively frequent condition, and that every gynæcologist in active practice must meet with several cases in the course of every year.

We now can better recognise this condition, and many of the deaths formerly put down to idiopathic peritonitis and hæmatocele were really deaths from ruptured tubal pregnancy. The causation of this trouble is now generally admitted to be some lesion or other of the tube causing an obstruction to the passage of the ovum. With the exception of Bland Sutton and one or two others, this is freely admitted, and it is quite the easiest explanation of the condition. It may occur at any age, in the first pregnancy a few months after marriage, or it may happen in a woman who has borne several children; the only condition necessary to precede it is some mischief in one or other of the tubes.

Now from the time an impregnation occurs in a Fallopian tube until that tube is removed, the patient is never free from danger. Hence, directly the diagnosis is made, the abdominal cavity should be opened and the whole thing removed. During the period prior to rupture, the operation is one of the simplest

[1] Read at the March Meeting of the Bristol Medico-Chirurgical Society.

in abdominal surgery, and in the hands of a skilled operator the mortality should be *nil;* in the second case, where rupture has taken place, the only chance for the patient is operation, and this should be performed at the earliest possible moment.

The following case well illustrates the importance of early operation:—

E. L., 27, was married at the age of 19, and had her first child before she was 20; since then she has had four children, and one miscarriage between the second and third children. The last child was born on September 3rd, 1896. There is a probable history of gonorrhœa after the birth of the first child; at any rate, there was a profuse yellow discharge, accompanied by swelling of the vulva and painful micturition.

In November patient had her ordinary period, and was apparently quite well until December 24th (no period in December). On December 24th she had acute abdominal pain and fainted, and from this date up to February 1st she remained in bed at home, and on the average she fainted three times a week, occasionally being suddenly seized with the pain in the night.

She states that the pain seized her on February 1st, at 8 a.m., just as the bowels were acting; she fainted and became very blanched, and Mr. Perry, the Medical Officer of the Clifton Dispensary, immediately brought her up to me at the Hospital.

On admission patient was extremely blanched, with a quick running pulse of 130. The abdomen was very rigid, but absolutely nothing could be felt, and *per vaginam* there was only an indistinct sense of resistance in the pouch of Douglas. There was no uterine discharge at all, nor had there been any.

At 11.30, or only three and a half hours after the fainting attack, I opened the abdomen in the middle line: on incising the peritoneum a great gush of blood took place, and it was evident that active hemorrhage was still proceeding. I ran my fingers quickly down on to the left tube, and passed over them a large pair of catch forceps and firmly clamped the left tube: active hemorrhage then ceased. On pulling up the left tube to the surface the rupture was easily seen, and the mass with the placenta was easily detached from the omentum and pulled out of the wound, but the fœtus was not to be seen. On exploring with the finger again a rounded mass was felt, lower down behind the pubes: on bringing this to the surface it was found to be the fœtus (probably about the ninth or tenth week) coiled up in a fold of the omentum; this was quickly released and extracted. The tube was then transfixed close to the uterus, and ligatured and removed, also a piece of omentum which appeared somewhat damaged. The operation had so far only taken twelve minutes, but now it was found that the whole pelvis was full of clot and fluid dark blood: this was gradually washed out with hot boracic lotion by means of a long glass tube carried down into the pouch of Douglas, and everywhere amongst the intestines; and there was such a large quantity that it took another twenty minutes to thoroughly cleanse the peritoneal cavity. A large glass drainage tube was passed into Douglas's pouch and left there, and the edges of the wound brought together by three sutures.

There was considerable shock, and the pulse was very quick and feeble. Directly the patient was put to bed she had an enema of three

12

ounces of hot port wine. From this time she slowly recovered, and had no bad symptom. I removed the glass drainage tube on the third day. On the twenty-first day after the operation she was able to get up in the ward. She is now quite well.

The operation was clearly the only chance of saving the patient's life: but at the time it was thought that the chance of recovery was very small, on account of the enormous amount of clot and fluid blood in the peritoneal cavity.

---

After the reading of this paper, the PRESIDENT said that the important point in these cases is an early diagnosis; this requires a great amount of skill in vaginal examination, as it is very difficult to tell the exact nature of retro- or peri-uterine swellings. He had seen a woman seven weeks pregnant, who was suddenly seized with acute abdominal pain and faintness as a result of lifting a heavy cask. Vaginal examination revealed a large post-uterine swelling and an enlarged uterus; within twelve hours the os uteri dilated, and an ovum was recognised protruding through the internal os uteri. This clearly proved that the pregnancy was intra-uterine. The woman aborted. The post-uterine swelling was an ordinary hæmatocele and became absorbed. The President also mentioned other cases bearing on the subject.—Dr. WALTER SWAYNE drew attention to the fact that in these cases a definite history of pregnancy is often absent. He described the case of a lady who habitually suffered from dysmenorrhœa, and was seized at the time of her period with acute abdominal pain, leaving collapse and fainting. From this she recovered, to be seized a month later with a similar attack of much greater gravity, which ended in death. The most probable explanation of this was that the two attacks of pain were due to the primary and secondary rupture of a tubal gestation. A case with this history, and showing signs of collapse and hemorrhage, can often only be successfully dealt with by abdominal section, and the gravity of such symptoms should always be kept in view.

---

